# Biomechanical comparisons of F.E.R.I. techniques with different type of intramedullary screws fixation for Jones fractures

**DOI:** 10.3389/fbioe.2024.1389127

**Published:** 2024-05-01

**Authors:** Kuo-Chih Su, Yu-Chun Yen, Chun-Hsiang Wang, Yi-Lun Wang, Shun-Ping Wang

**Affiliations:** ^1^ Department of Medical Research, Taichung Veterans General Hospital, Taichung, Taiwan; ^2^ Department of Medical Equipment Development and Application, HungKuang University, Taichung, Taiwan; ^3^ Stella Matutina Girls’ High School, Taichung, Taiwan; ^4^ Department of Orthopaedics, Taichung Veterans General Hospital, Taichung, Taiwan; ^5^ Department of Post-Baccalaureate Medicine, College of Medicine, National Chung Hsing University, Taichung, Taiwan

**Keywords:** Jones fracture, biomechanics, screw, maximal force, stiffness, headless screws, SG-HCS, F.E.R.I

## Abstract

**Introduction:** Jones fractures frequently fail to unite, and adequate fixation stability is crucial. This study aimed to elucidate the biomechanical stability of various intramedullary screw fixation constructs.

**Methods:** Jones fracture model over the proximal 5th metatarsal of artificial bone was created in all specimens. Six groups were divided based on varied screw constructs with different screw lengths, either 30 or 40 mm, including cannulated screws—C30 and C40 groups, one high-resistance suture combined with intramedullary cannulated screws (F.E.R.I. technique)—CF30 and CF40 groups, and second-generation headless compression screws (SG-HCS) —HL30 and HL40 groups. Mechanical testing was conducted sequentially, and the maximal force (N) and stiffness (N/mm) of all constructs were recorded.

**Results:** The maximal force (N) at 1.0 mm downward displacement in C30, C40, CF30, CF40, HL30, and HL40 groups were 0.56 ± 0.02, 0.49 ± 0.02, 0.65 ± 0.02, 0.49 ± 0.01, 0.68 ± 0.02, and 0.73 ± 0.02, respectively, and the stiffness (N/mm) in subgroups were 0.49 ± 0.01, 0.43 ± 0.01, 0.67 ± 0.01, 0.42 ± 0.01, 0.61 ± 0.01, and 0.58 ± 0.02, respectively. SG-HCS subgroups exhibited greater maximal force and stiffness than conventional cannulated screws. Screws of 30 mm in length demonstrated better stability than all 40 mm-length screws in each subgroup. In C30 fixation, the stiffness and maximum force endured increased by 1.16 and 1.12 times, respectively, compared with the C40 fixation method. There were no significant differences between CF30 and SG-HCS groups. Only the F.E.R.I technique combined with the 4.5 mm cannulated screw of 30 mm in length increased the biomechanical stability for Jones fractures.

**Discussion:** These biomechanical findings help clinicians decide on better screw fixation options for greater stability in Jones fractures, especially when large-diameter screws are limited in use. However, this biomechanical testing of intramedullary screw fixation on Jones fracture model lacks clinical validation and no comparisons to extramedullary plate fixations. Moving forward, additional clinical and biomechanical research is necessary to validate our findings.

## 1 Introduction

Jones fractures, located in zone 2 of the proximal fifth metatarsal (MT-5) at the metaphyseal–diaphyseal junction, frequently occur in elite athletes (Dean et al., 2012; Roche and Calder, 2013). Jones fractures make up 17.8% of all foot fractures in National Football League (NFL) players, (Kavanaugh et al., 1978) which is significantly higher than the reported incidence of all foot fractures in the general population, ranging from 0.7% to 1.9% (Josefsson et al., 1994). The limited blood supply within a vascular watershed area may be interrupted at the fracture site, subsequently leading to delayed union or non-union of fractures (Bluth et al., 2011; McKeon et al., 2013). Treatment options for Jones fractures vary; however, non-operative treatment carries a higher risk of non-union, with reported failure rates of 21%–44% after cast immobilization (Mologne et al., 2005; Chloros et al., 2022) and prolonged healing times compared to surgical interventions (Wheeler and McLoughlin, 1998; Roche and Calder, 2013; Yates et al., 2015). Therefore, surgical management is recommended for Jones fractures to prevent subsequent nonunion of fractures and refracture (Porter, 2018; Singh et al., 2018; Attia et al., 2021).

Surgical fixation using implants, including intramedullary screws, tension band wiring, or extramedullary plates, has been proposed to stabilize proximal MT-5 fractures for bony healing, yielding favorable surgical outcomes (Lee et al., 2011; Massada et al., 2012; Seidenstricker et al., 2017; Varner and Harris, 2017; Goh et al., 2018). Intramedullary screw fixation of Jones fractures offers advantages such as ease of application and cost-effectiveness compared to plate fixation. Therefore, intramedullary fixation with partially threaded cannulated screws has traditionally been the implant of choice for Jones fractures, allowing athletes early weight-bearing and return to sport. However, conventional cannulated screw fixation, especially with small diameters, may inherently struggle to resist loads from peroneus brevis pulling (Willegger et al., 2020b), resulting in failure rates of 5%–7.3% (Wright et al., 2000; Granata et al., 2015). Headless Herbert-style compression screws and other fixation techniques aimed at achieving superior initial stability have been introduced to reduce failure rates (Lee et al., 2011; Lam et al., 2021).

Larger diameter intramedullary screw fixation in Jones fractures has been reported to improve stability and enhance union rates (Willegger et al., 2020a). However, the diameter of the screw will be constrained by the limited inner diameter of the curve-shaped medullary canal of MT-5 for Jones fractures (Orr et al., 2012). Furthermore, the MT-5 medullary canal is not straight, and the coronal diameter is less than 4.5 mm in 19% of males and 26% of females, respectively (Ochenjele et al., 2015). Therefore, modified screw designs or reinforcement with suture cerclage on conventional cannulated screws were introduced to enhance initial stability for Jones fractures. D’Hooghe et al. proposed a new modified fixation method with a combination of a cannulated screw and additional high resistance suture cerclage of MT-5 fractures, named the F.E.R.I. technique (D’Hooghe et al., 2019). They believed the F.E.R.I. technique offers superior stability in fixation for proximal MT-5 fractures, but there were no biomechanical comparisons with other fixation methods in their study.

Moreover, second-generation headless compression screws (SG-HCS), which are headless, tapered, continuously threaded screws with variable pitches, have been developed to improve compression force on fracture sites to enhance bony union. Previous mechanical evaluations in the scaphoid showed SG-HCS to have equal or superior compression force compared to Herbert-style screws (Beadel et al., 2004; Haisman et al., 2006; Hausmann et al., 2007; Assari et al., 2012) but there was no significant improvement in stability of intramedullary SG-HCS compared to conventional screws for Jones fractures (Sides et al., 2006; Orr et al., 2012).

The above literature review focuses on Jones fractures and elucidates the pros and cons of different treatment strategies, introduces the F.E.R.I. technique, and discusses studies related to headless compression screws. The majority of previous research on Jones fractures has only focused on intramedullary fixation with different screw diameters or designs and their surgical outcomes clinically. However, there has been few biomechanical assessment of different screw fixation methods on Jones fracture. The comparisons of biomechanical stability between SG-HCS and conventional cannulated screws for Jones fractures are still controversial. The verification of biomechanical stability of intramedullary conventional screws, F.E.R.I., and SG-HCS constructs for Jones fractures is still scarce. Therefore, our findings in this study will provide biomechanical evidence of various screw fixations for Jones fractures, particularly aiding clinicians in deciding optimal fixation techniques for cases with a small diameter of the intramedullary canal of MT-5.

The purpose of this study is to compare the biomechanical stability of different constructs of intramedullary screw fixation, including conventional cannulated screws, the F.E.R.I. technique, and SG-HCS, with varied screw lengths, respectively, on a Jones fracture model. We hypothesize that the F.E.R.I. technique can improve the stability of conventional cannulated screws and be equivalent to that of SG-HCS.

The novelty of this study:

• Investigate the biomechanical conditions of different intramedullary screw fixation methods, including conventional screws, the F.E.R.I. technique, and SG-HCS constructs, on Jones fractures.
• Verify whether the F.E.R.I. technique indeed enhances the stability of Jones fracture fixation.

## 2 Materials and methods

### 2.1 Specimen preparation

To simulate Zone 2 fractures of the proximal 5th metatarsal bone, artificial feet bones (9147, SYNBONE, Zizers, Switzerland) were utilized in this research. Biomechanical testing of various fixations on the stability of proximal 5th metatarsal fractures was conducted. Following the simulation of fractures on the proximal 5th metatarsal bone and fixation using different implants according to the assigned groups, the distal parts of the repaired 5th metatarsal bones were embedded in poly resin. Specimens were then mounted and secured in the designed fixtures attached to a material test system (MTS) for further biomechanical testing.

### 2.2 Fracture creation and screw fixation

**FIGURE 1:**
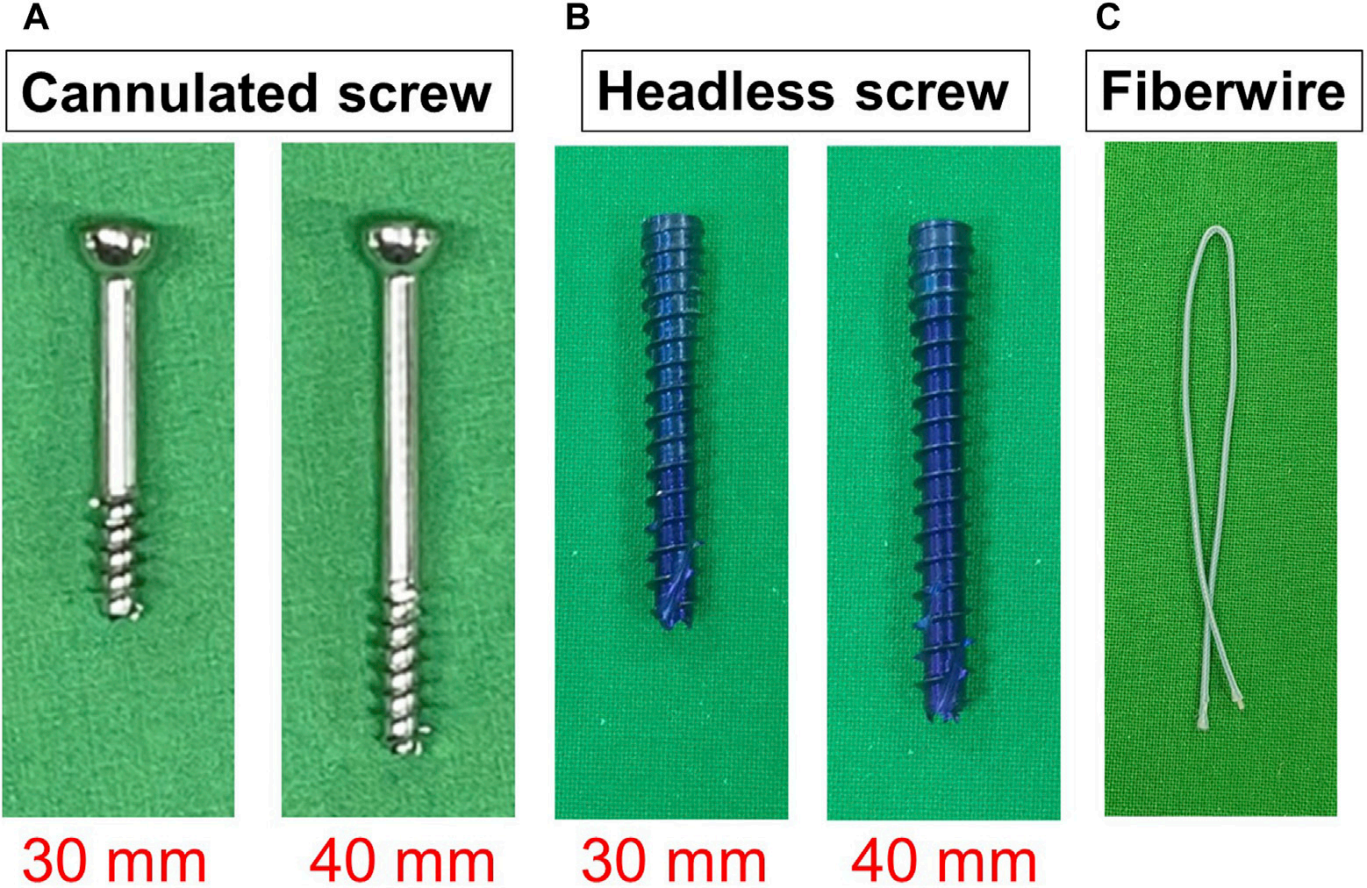
Implants of fracture fixation. **(A)** 4.5-mm stainless partially-threaded cannulated screws. **(B)** 4.7 mm Acutrak-2 screw. **(C)** Fiberwire.

An oblique linear fracture directed towards the 4th-5th metatarsal articulation was created on a precision position table using a saw at a distance of 1.5 cm from the proximal tuberosity of the 5th metatarsal. The entrance of screws was made through the guided wire placed into the intramedullary canal by drilling from the tip of the tuberosity of the proximal 5th metatarsal. The fractures at the proximal 5th metatarsal were fixed using partially headed cannulated or tapered headless screws commonly used for fractures of the 5th metatarsal. Screws included 4.5 mm stainless steel partially-threaded cancellous screws (Synthes, United States, Monument, CO) and 4.7 mm Acutrak-2 screws (Acumed, Beaverton, OR, United States). The Acutrak-2 screw is tapered, with variable pitch titanium screws having a leading thread diameter of 4.5 mm and a trailing thread diameter of 4.7 mm. Both 30 mm and 40 mm screws in length were used in both screw groups, respectively (Figure 1).

**FIGURE 2:**
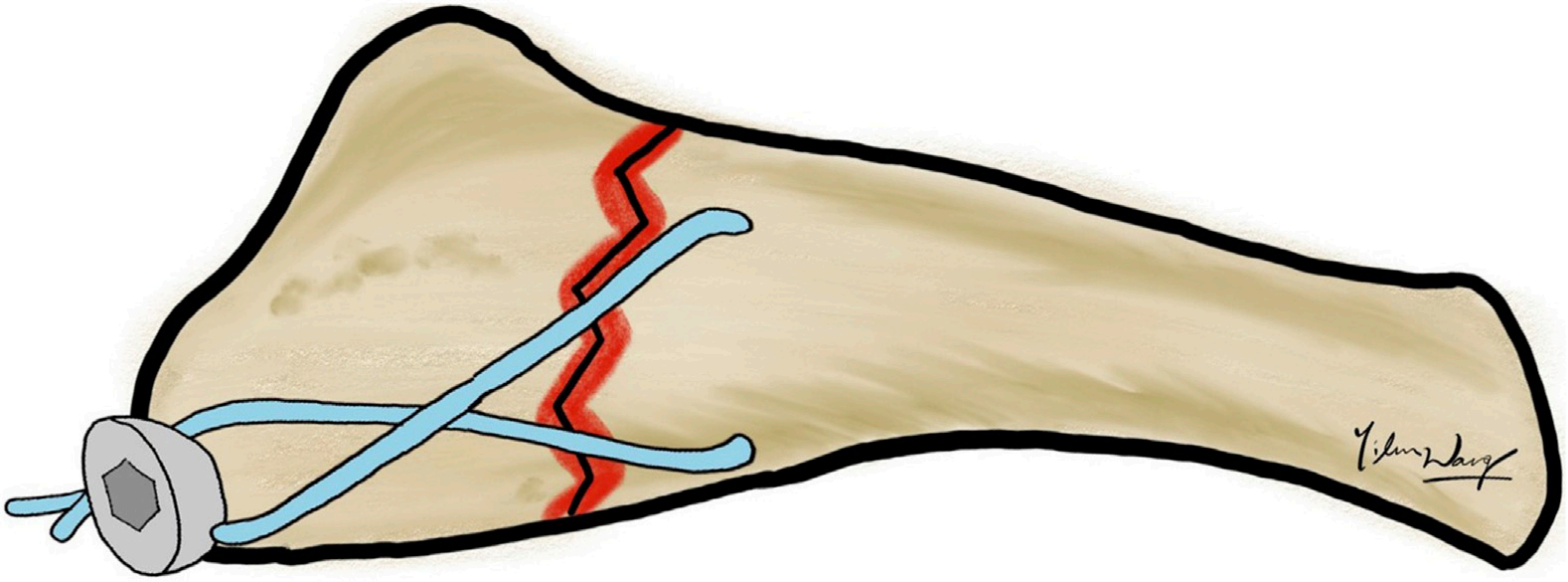
F.E.R.I. technique. A no. 2 Fiberwire was passed through the through-hole tunnel in a figure-of-eight pattern combined with one intramedullary cannulated screw.

Additionally, in the cannulated screw fixation group, a high-resistance suture, Fiberwire (Arthrex, Naples, FL, United States), was further used to augment the stability of screw fixation using the F.E.R.I. technique (D’Hooghe et al., 2019). Briefly, a bony through-hole tunnel was drilled vertically 10 mm distal to the fracture line over the proximal 5th metatarsals. A no. 2 Fiberwire was passed through the tunnel and then pulled from the distal to the proximal in a figure-of-eight pattern. Finally, it was securely knotted around the neck of the headed cannulated screw. The screw was placed along the guide wire and then tightened, and manual maneuvers were performed to check the final fracture stability. Ultimately, the distal part of the 5th Metatarsal bone was embedded in poly resin for further mechanical testing (Figure 2).

### 2.3 Experimental groups

**FIGURE 3:**
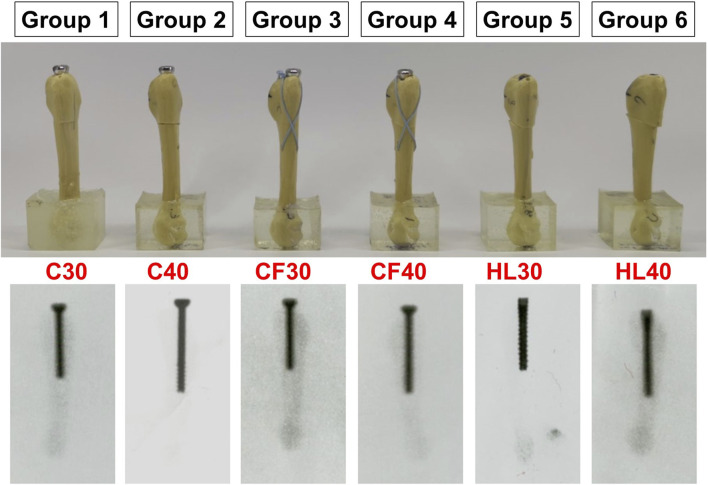
The testing groups according to the different fixating methods of Jones fractures.

According to the fixation constructs for of Jones fracture, there were a total of 6 groups included on the basis of different screw types, including partially-threaded cannulated screw, F.E.R.I. and tapered SG-HCS, and screw length, either 30 mm or 40 mm in length (Figure 3) Each groups have 10 biomechanical tests for each 0.1 mm increment of downward displacement at fracture sites.

Group 1 (C30): Fixed by 30 mm partially-threaded cannulated screw
Group 2 (C40): Fixed by 40 mm partially-threaded cannulated screw
Group 3 (CF30): Fixed by 30 mm partially-threaded cannulated screw + F.E.R.I
Group 4 (CF40): Fixed by 40 mm partially-threaded cannulated screw + F.E.R.I
Group 5 (HL30): Fixed by 30 mm tapered SG-HCS
Group 6 (HL40): Fixed by 40 mm tapered SG-HCS

### 2.4 Biomechanical testing

**FIGURE 4:**
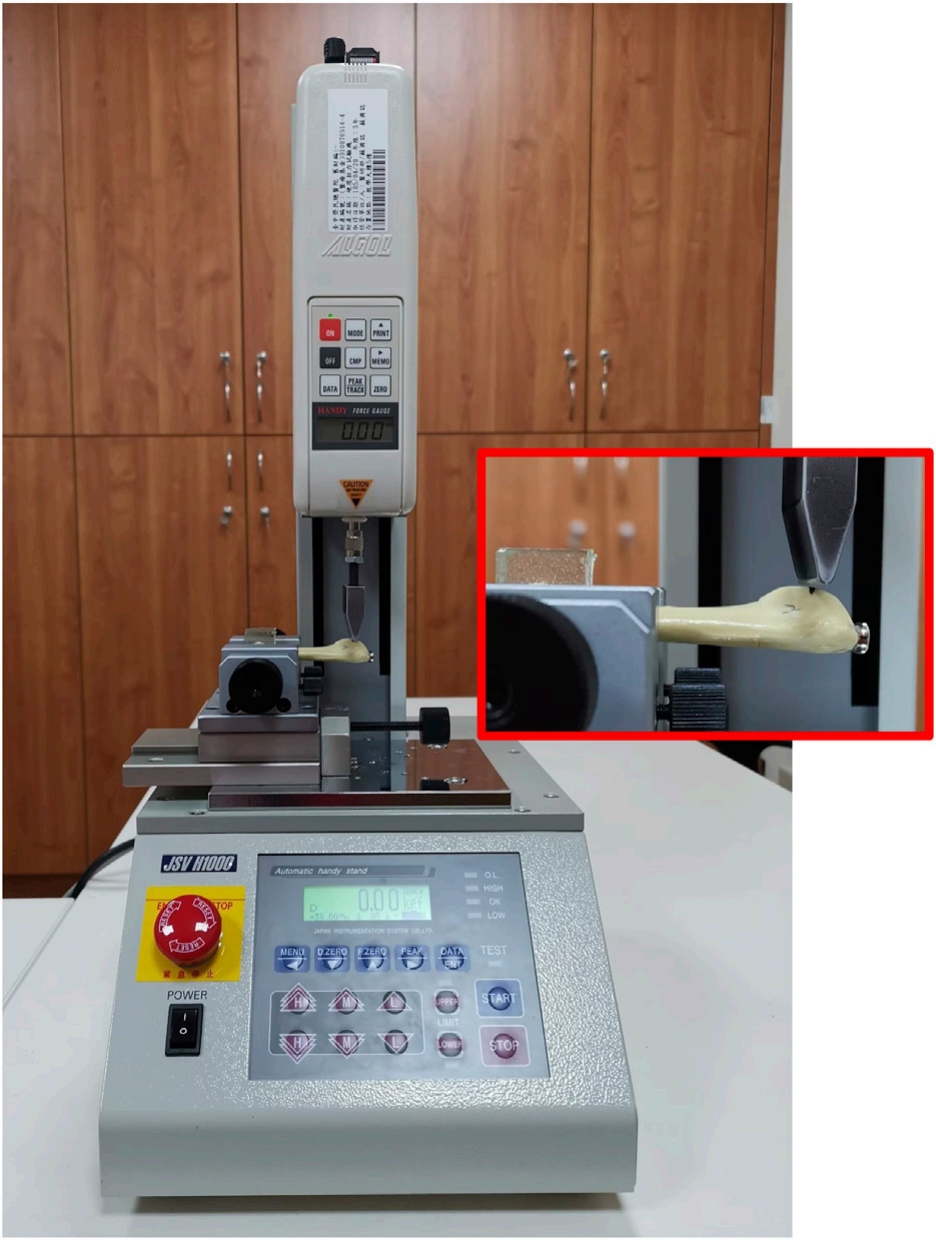
Biomechanical testing. The distal part of the 5th metatarsal bone was mount in the fixture of MTS and the cantilever loaded the repaired bone using various fixation constructs to displace downward the fracture site.

To simulate the non-destructive loads on the fractured bone after surgical fixation in a clinical scenario, submaximal loads rather than maximal loads to failure were applied to the experimental specimens in this study. These tests elucidated the variations in construct stability with increasing loads and analyzed the differences in mechanical stiffness and force at 1 mm displacement at the fracture site among various fixation methods. The distal end of the 5th artificial metatarsal was embedded in poly resin and mounted in the fixture of the MTS (JSV-H1000, Japan Instrumentation System, Nara, Japan) (Figure 4).

The cantilever was positioned 1 cm away from the proximal tip of the 5th metatarsal, and downward load was applied to the ground for a bending test from the plantar aspect of the 5th metatarsal base. All samples in the experimental groups were preloaded to stabilize the cantilever on the bone. The bone was loaded to displace the fracture site with various fixation groups from 0.1 mm to 1.0 mm, with a 0.1 mm increment each time. Ten rounds of each testing were performed. Displacement-force data were recorded during the experiment, and the stiffness (N/mm) of fixation constructs was determined. The maximal forces (N) on the MTS on the 5th Metatarsal bone at 1.0 mm displacement of fractures were also recorded to determine the strength of fixation constructs.

### 2.5 Statistical analysis

The continuous data was presented with means ± standard deviation (SD). The categorical data were presented with frequencies and percentages. The normality of continuous data was checked by Kolmogorov-Smirnov test. The maximum force and stiffness of the different fixation approaches were analyzed using Kruskal-Wallis test by Bonferroni test with a 0.05 level of significance. All statistical analyses were performed using the Statistical Package for the Social Science (IBM SPSS version 22.0; International Business Machines Corp, New York, United States). The level of statistical significance was set at *p* < 0.05. In this study, ANOVA was employed to test for mean differences. Therefore, the null hypotheses state that the means of the six experimental groups (C30, C40, CF30, CF40, HL30, and HL40) are equal, while the alternative hypotheses suggest rejecting the null hypotheses, indicating that at least one group’s mean differs from the others.

## 3 Results

The maximal force (mean ± SD) at 1.0 mm displacement of the fracture site in the C30, C40, CF30, CF40, HL30, and HL40 groups were 0.56 ± 0.02 N, 0.49 ± 0.02 N, 0.65 ± 0.02 N, 0.49 ± 0.01 N, 0.68 ± 0.02 N, and 0.73 ± 0.02 N, respectively. The required loads in the CF30, HL30, and HL40 groups were higher than in the C30, C40, and CF40 groups. Regarding stiffness, the stiffness (mean ± SD) in the C30, C40, CF30, CF40, HL30, and HL40 subgroups were 0.49 ± 0.01 N/mm, 0.43 ± 0.01 N/mm, 0.67 ± 0.01 N/mm, 0.42 ± 0.01 N/mm, 0.61 ± 0.01 N/mm, and 0.58 ± 0.02 N/mm, respectively. There was higher stiffness in the CF30, HL30, and HL40 groups compared to the C30, C40, and CF40 groups. The results revealed higher maximal force and stiffness of the constructs in the SG-HCS groups and 30 mm cannulated screw with Fiberwire reinforcement. The differences in both maximal force and stiffness between subgroups reached statistical significance (Table 1).

**TABLE 1:** Maximal force and Stiffness of various fixations.

	C30	C40	CF30	CF40	HL30	HL40	*p*-Value
Max Force (N)							
mean (SD)^a^	0.56 ± 0.02	0.49 ± 0.02	0.65 ± 0.02	0.49 ± 0.01	0.68 ± 0.02	0.73 ± 0.02	<0.001**
median^b^	0.56	0.50	0.65	0.49	0.73	0.69	<0.001**
IQR	0.54–0.57	0.48–0.51	0.64–0.67	0.49–0.50	0.72–0.75	0.67–0.69	
Stiffness (N/mm)							
mean (SD)^a^	0.49 ± 0.02	0.43 ± 0.01	0.58 ± 0.02	0.42 ± 0.01	0.61 ± 0.01	0.67 ± 0.01	<0.001**
median^b^	0.50	0.43	0.60	0.42	0.66	0.61	<0.001**
IQR	0.48–0.50	0.42–0.44	0.56–0.60	0.42–0.43	0.65–0.68	0.60–0.62	

^a^ ANOVA.

^b^ Kruskal-Wallis test. *p < 0.05, **p < 0.01.

**FIGURE 5:**
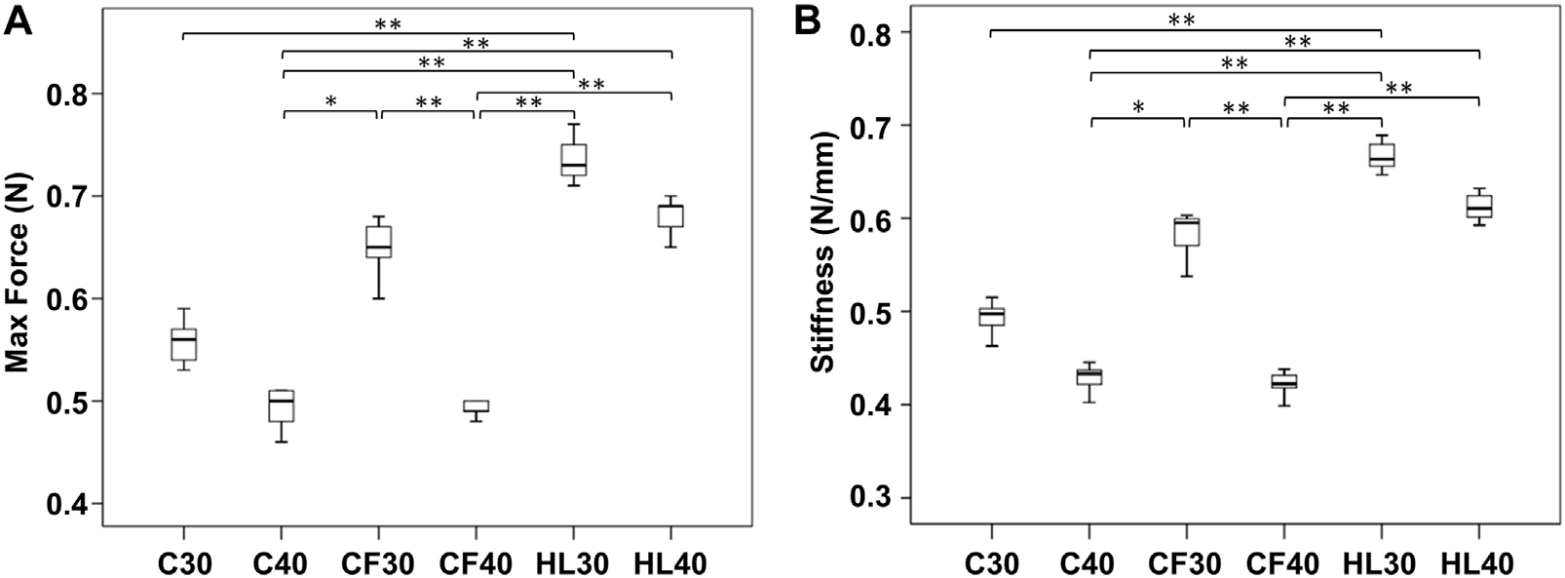
The statistical comparisons between subgroups **(A)** Maximal force (N) at 1 mm displacement downward of fixation constructs and **(B)** Stiffness (N/mm).

After *post hoc* analysis, the maximal force and stiffness of subgroups were demonstrated by box plots, respectively (Figures 5A, B). Both the maximal force and stiffness of SG-HCS groups (HL30, HL40) were significantly higher than the cannulated screw groups (C30, C40) (*p* < 0.001). The maximal force and stiffness of SG-HCS were also higher than CF40 (*p* < 0.001), but there were no significant differences between the SG-HCS group and CF30 (HL30 vs. CF30, *p* = 1.0; HL40 vs. CF30, *p* = 0.234). Furthermore, in the cannulated screw, F.E.R.I., and SG-HCS subgroups, the maximal force and stiffness of screws with 30 mm in length were significantly higher than 40 mm screws. According to the results, under the same screw constructs, longer screws did not demonstrate more biomechanical stability for fractures.

**FIGURE 6:**
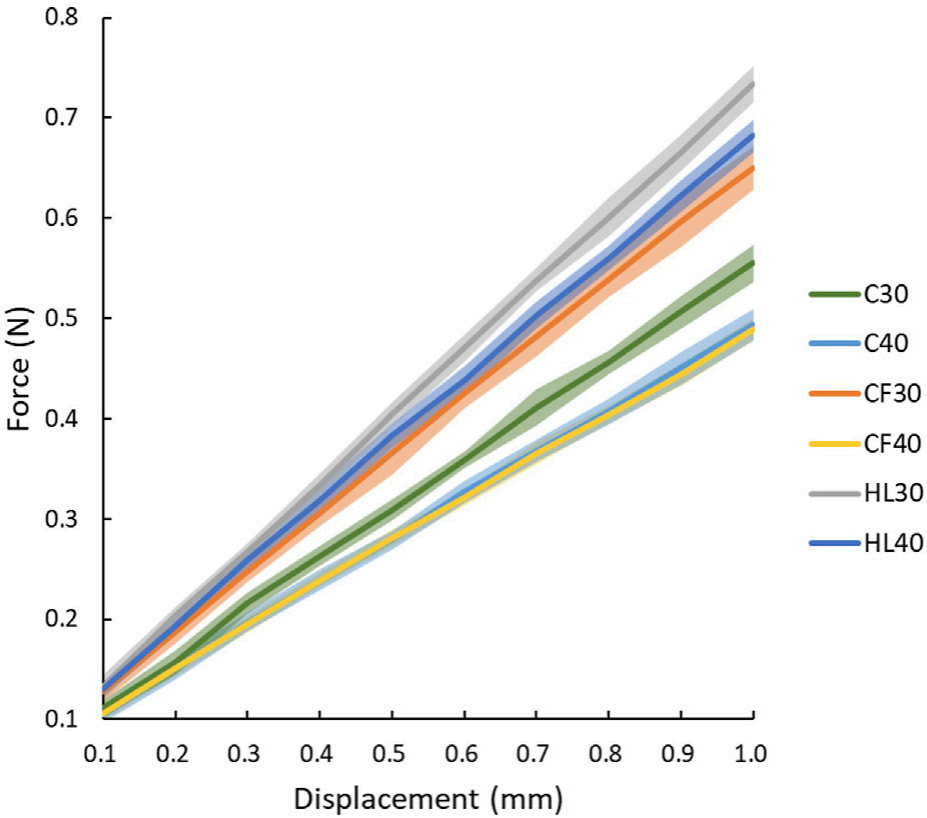
The force versus displacement for mechanical tests on different fixations.

The Figure 6 demonstrated the force versus displacement for mechanical tests with 0.1 mm increment of fracture sites under loading in different fixation constructs. The most steep slop, i.e. stiffness, was in HL30 group, in order, the following were HL40, CF30, C30, C40, and CF40.

## 4 Discussion

In this study, we utilized an artificial bone fracture model to verify the biomechanical stability of diverse constructs of medium-diameter screw for Jones fractures. SG-HCS subgroups exhibited greater maximal force and stiffness than conventional cannulated screws. Screws of 30 mm in length demonstrated better stability than all 40 mm-length screws in each subgroup. With a 30-mm length conventional screw fixation (C30), both stiffness and maximum force endurance increased by 1.16 and 1.12 times, respectively, in comparison to the C40 fixation. Only the F.E.R.I. technique, when combined with a 4.5 mm cannulated screw measuring 30 mm in length, resulted in a biomechanical stability equivalent to that of fixation with SG-HCS for Jones fractures.

Jones fracture, characterized as a Zone 2 fracture of the proximal 5th metatarsal, is notorious for nonunion, malunion, and refracture following surgical fixation, particularly in elite athletes. Intramedullary screw fixation, with its relatively minimally invasive surgical approach, offers potential advantages such as reduced wound complications and irritations. However, concerns persist regarding the inherent biomechanical instability associated with fracture fixation. Although SG-HCS has been touted for its biomechanical stability in enhancing fracture union rates (Kibar et al., 2022; Wallace et al., 2023), its application in Jones fractures using medium-diameter tapered headless screws remains controversial (Demel et al., 2023). Additionally, the F.E.R.I. technique, involving Fiberwire suture augmentation with cannulated screw fixation, has been introduced to enhance fracture stability, but its clinical and biomechanical benefits require further investigation. To our knowledge, we are the first to compare the mechanical stability among conventional cannulated screws, the F.E.R.I technique, and SG-HCS for Jones fractures.

Based on previous reports, approximately one-fifth of the population has an intramedullary diameter below 4.5 mm and curve-shaped intramedullary canal (Ochenjele et al., 2015). These factors prevent the usage of large-diameter intramedullary screw fixation for Jones fracture. In this current study, we specifically utilized medium-diameter screws, including 4.5 mm cannulated screws and 4.7 mm Acutrak-2 screws, to fix the Jones fracture model and compared their biomechanical strength. We found that SG-HCS offered significantly more biomechanical stability than conventional cannulated screws with varied screw lengths, either 30 mm or 40 mm. However, the significant increase in maximal force and stiffness was only found in the 30 mm cannulated screw with Fiberwire reinforcement, not in the 40 mm cannulated screw. The biomechanical stability of Fiberwire augmented with 30 mm cannulated screw (CF30 group) was equivalent to that of the SG-HCS groups. Our experimental results show that screws of appropriate length and using Fiberwire reinforcement can increase biomechanical strength by 1.2 times compared to intramedullary screw fixation alone.

**FIGURE 7:**
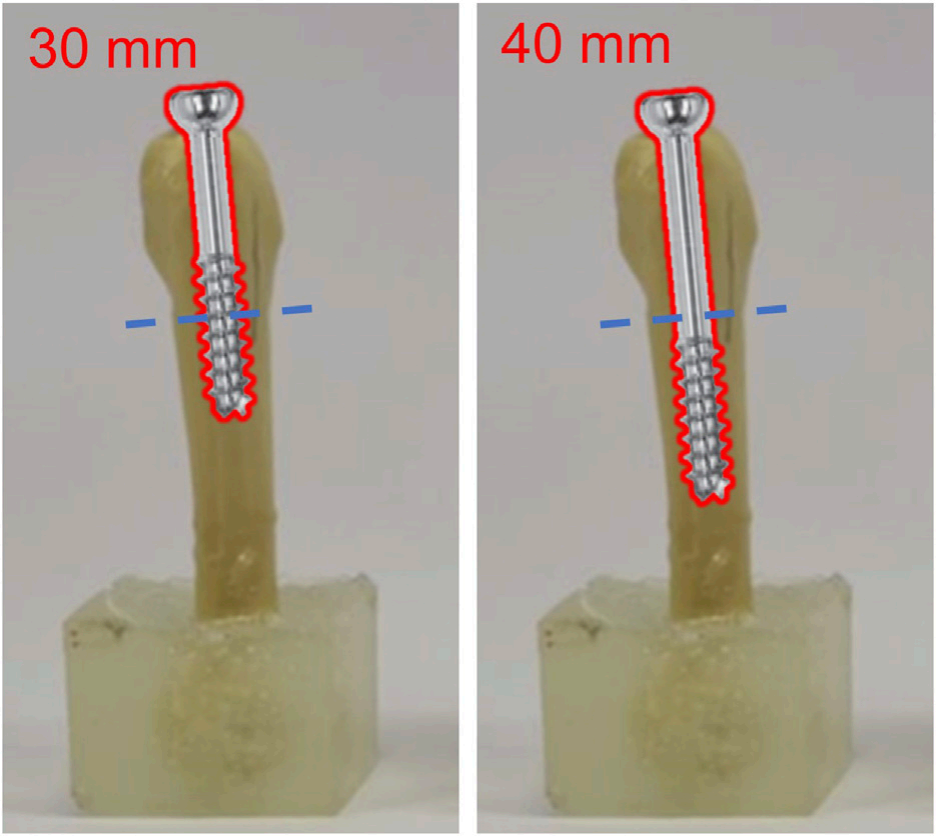
The relations between intramedullary cannulated screws with varied length and the fracture site of Jone fracture.

Furthermore, when utilizing cannulated screws of different lengths, either 30 mm or 40 mm, for Jones fracture fixation, it was observed that longer cannulated screw implantation did not result in increased stiffness. Greater stiffness indicates a more stable fixation effect, as the 5th metatarsal proximal fracture is subjected to the same displacement, requiring a greater force to achieve the same displacement. In partially threaded cannulated screw fixations, this study found that using the C30 fixation method resulted in a stiffness increase of 1.16 times compared to the C40 fixation method (the maximum force endured could be increased by 1.12 times). The main reason for this might be that the fracture site of the Jones fracture was wedged onto the screw threads (Figure 7). Consequently, the screw threads could effectively stabilize the upper and lower ends of the fracture, thereby providing better stability. We found that longer screws did not necessarily provide greater stability, and appropriate screw length also influenced fixation stability. This finding in our study has not yet been reported in the fixation of Jones fractures, but it is consistent with reports on metacarpal fractures by Yamaguchi et al. (Yamaguchi Jr et al., 2023). Therefore, when selecting screw length for 5th proximal metatarsal bone fractures, attention should be paid to the location of the fracture.

In addition to selecting a screw with appropriate length for fixation of Jones fractures, D’Hooghe et al. suggested that the F.E.R.I technique, involving fixation with an intramedullary screw combined with a high-resistance suture, provides increased stability for Jones fractures (D’Hooghe et al., 2019). In this study, intramedullary cannulated screws combined with Fiberwire were found to increase the stiffness of Jones fracture fixation. However, biomechanical strength was only enhanced when a high-resistance suture was augmented with a 30 mm cannulated screw, but not with a 40 mm screw. This suggests that the increase in stiffness was not solely attributed to the combination of cannulated screw and Fiberwire, but also depended on matching the correct cannulated screw length. The combination of the F.E.R.I technique with an appropriate screw length allows the 5th metatarsal bone to withstand greater maximal force.

**FIGURE 8:**
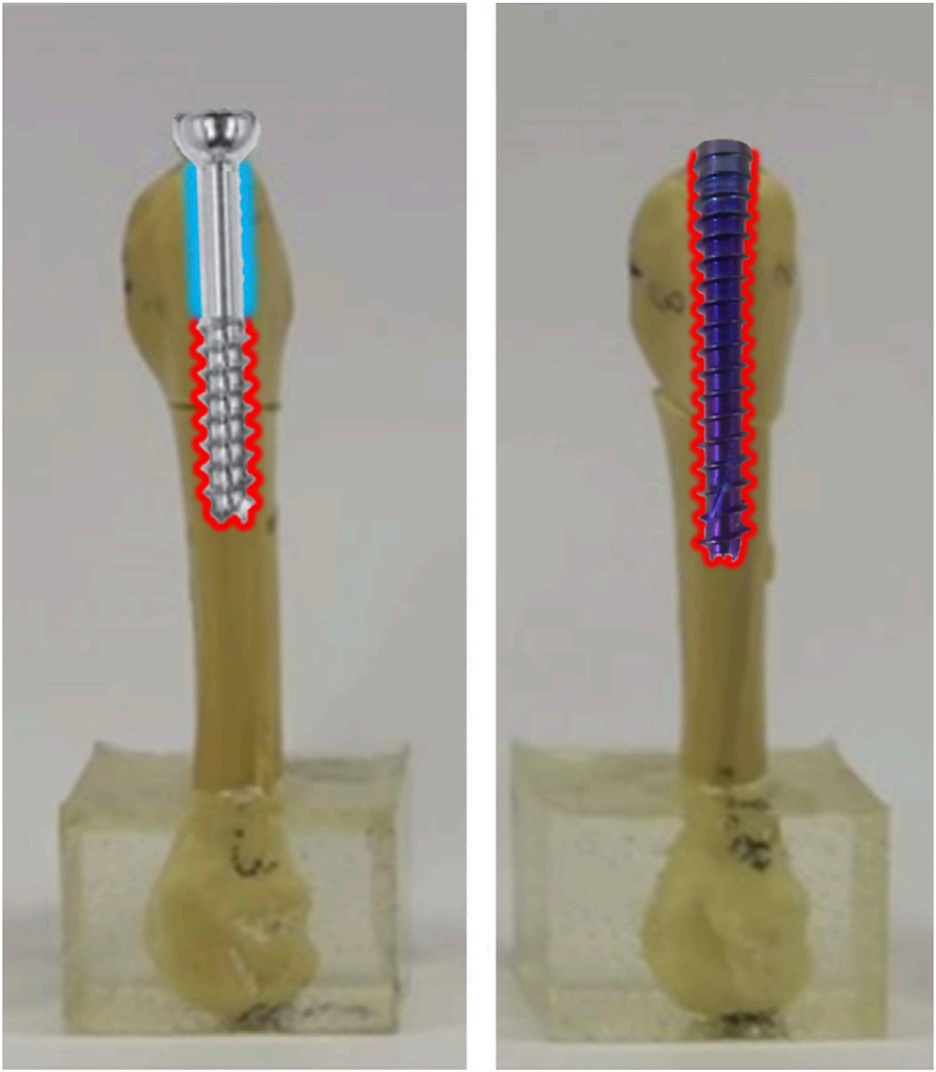
The comparisons of fixation area by threads using partially threaded cannulated screws and SG-HCS in Jones fracture.

According to the results of this study, the stiffness of tapered headless screw fixation is higher than that of cannulated screw stabilization. The main reason for this difference may be attributed to the design and mechanics of the screws. When a cannulated screw of appropriate length is used for fixation, although the threads effectively fix the upper and lower bones of the fracture (as indicated by the red area in Figure 8), there will be a smooth surface area where the screw has no thread (the blue area in Figure 8). In contrast, the SG-HCS has a thread-fixed area (the red area in Figure 8) that is more extensive compared to the conventional cannulated screw. Additionally, the thread design of SG-HCS, with continuous varied pitch, increases compression force at the fracture site, resulting in higher stiffness. Therefore, compared to conventional cannulated screws, the usage of SG-HCS also provides higher stability for Jones fractures.

Some previous articles have reported that the fixation effect of SG-HCS is not better than that of partially threaded cannulated screws (Sides et al., 2006; Orr et al., 2012). However, these studies often utilized cadaveric bones, which exhibit individual differences, leading to variations in the size of the 5th metatarsals, particularly in cases with smaller body sizes. Consequently, the authors were unable to use screws with the same larger diameter to fix Jones fractures in their research. Instead, they selected the diameter of the fixation screw based on the estimated inner diameter of the bone canal during the experiment, which may have introduced biases into the biomechanical comparisons. In contrast, in this current study, we utilized synthetic bones (synbones) instead of cadaveric bones, which offer consistent quality and the advantage of homogeneity. However, it is important to note that the results reported herein still require further research to confirm our findings.

There are several limitations in this study that should be acknowledged. Firstly, there was no correlation with clinical outcomes in this research, which could have provided valuable insights into the practical implications of the biomechanical findings. Moreover, the use of artificial bones instead of fresh human 5th metatarsal bones may limit the generalizability of the results, as artificial bones cannot fully replicate the characteristics of real human bones, including anisotropy and trabecular bone structure. Additionally, the evaluation of stability was conducted solely through cantilever bending testing on the 5th metatarsal bone alone, which may not fully represent the complex loading conditions experienced *in vivo*. Furthermore, the mechanical experiments in this study did not apply loads to failure of the fixation constructs on the 5th metatarsal bone. While this was a deliberate choice to focus on exploring the biomechanical fixation strength under varying load conditions at the fracture site, it is important to note that clinical suboptimal loading conditions may not generally result in screw failure.

## 5 Conclusion

The intramedullary cannulated medium-diameter screw combined with a high resistance suture, such as Fiberwire, increased the biomechanical stability in terms of stiffness and maximal force of fixation for Jones fractures and may be a viable solution to enhance fixation strength if the use of large-diameter screws is limited due to the small inward diameter of the medullary canal of the 5th metatarsal. However, screw length also influences the stability of fixation constructs on proximal 5th metatarsal fractures. A longer screw length does not necessarily increase fixation capability. Therefore, clinical utilization of the Fiberwire combined with the optimal screw length based on the fracture location is crucial for increasing biomechanical stability and to potentially enhance fracture union of Jones fractures. These findings provide scientific evidence to assist clinicians in selecting intramedullary screw fixation options for greater stability in Jones fractures, especially when the use of large-diameter screws is limited. Nevertheless, additional clinical and biomechanical research comparing F.E.R.I screw fixation with extramedullary plate fixation for Jones fractures is necessary to validate our findings.

## Data Availability

The original contributions presented in the study are included in the article/Supplementary material, further inquiries can be directed to the corresponding author.

## References

[B1] Assari S. Darvish K. Ilyas A. M. (2012). Biomechanical analysis of second-generation headless compression screws. Inj 43 (7), 1159–1165. 10.1016/j.injury.2012.03.015 22482931

[B2] Attia A. K. Taha T. Kong G. Alhammoud A. Mahmoud K. Myerson M. (2021). Return to play and fracture union after the surgical management of Jones fractures in athletes: a systematic review and meta-analysis. Am. J. Sports Med. 49 (12), 3422–3436. 10.1177/0363546521990020 33740393

[B3] Beadel G. P. Ferreira L. Johnson J. A. King G. J. (2004). Interfragmentary compression across a simulated scaphoid fracture—analysis of 3 screws. J. Hand Surg. 29 (2), 273–278. 10.1016/j.jhsa.2003.12.006 15043901

[B4] Bluth B. Eagan M. Otsuka N. Y. (2011). Stress fractures of the lateral rays in the cavovarus foot: indication for surgical intervention. Orthopedics 34 (10), e696–e699. 10.3928/01477447-20110826-28 21956071

[B5] Chloros G. D. Kakos C. D. Tastsidis I. K. Giannoudis V. P. Panteli M. Giannoudis P. V. (2022). Fifth metatarsal fractures: an update on management, complications, and outcomes. EFORT Open Rev. 7 (1), 13–25. 10.1530/eor-21-0025 35073515 PMC8788151

[B6] Dean B. J. F. Kothari A. Uppal H. Kankate R. (2012). The Jones fracture classification, management, outcome, and complications: a systematic review. Foot Ankle Spec. 5 (4), 256–259. 10.1177/1938640012444730 22547534

[B7] Demel J. Plánka L. Stichhauer R. Vrtková A. Bajor G. Havlicek M. (2023). 5 th metatarsal jones fracture–to treat conservatively, or surgically using headless double-threaded Herbert screw? Acta Chir. Orthop. Traumatol. cechoslov. 90 (1), 53–58. 10.55095/achot2023/008 36907584

[B8] D’Hooghe P. Caravelli S. Massimi S. Calder J. Dzendrowskyj P. Zaffagnini S. (2019). A novel method for internal fixation of basal fifth metatarsal fracture in athletes: a cadaveric study of the FERI technique (Fifth metatarsal, Extra-portal, Rigid, Innovative). J. Exp. Orthop. 6 (1), 45–47. 10.1186/s40634-019-0213-5 31713049 PMC6848546

[B9] Goh E. L. Chidambaram S. Eigenmann D. Ma S. Jones G. G. (2018). Minimally invasive percutaneous plate osteosynthesis versus intramedullary nail fixation for closed distal tibial fractures: a meta-analysis of the clinical outcomes. SICOT-J 4, 58. 10.1051/sicotj/2018055 30560779 PMC6298240

[B10] Granata J. D. Berlet G. C. Philbin T. M. Jones G. Kaeding C. C. Peterson K. S. (2015). Failed surgical management of acute proximal fifth metatarsal (Jones) fractures: a retrospective case series and literature review. Foot Ankle Spec. 8 (6), 454–459. 10.1177/1938640015592836 26130624

[B11] Haisman J. M. Rohde R. S. Weiland A. J. (2006). Acute fractures of the scaphoid. JBJS 88 (12), 2750–2758. 10.2106/00004623-200612000-00026 17219705

[B12] Hausmann J. Mayr W. Unger E. Benesch T. Vecsei V. Gäbler C. (2007). Interfragmentary compression forces of scaphoid screws in a sawbone cylinder model. Inj 38 (7), 763–768. 10.1016/j.injury.2006.11.002 17270187

[B13] Josefsson P. O. Karlsson M. Redlund-Johnell I. Wendeberg B. (1994). Jones fracture surgical versus nonsurgical treatment. Clin. Orthop. Relat. Res. 299, 252–255. 10.1097/00003086-199402000-00035 8119027

[B14] Kavanaugh J. H. Brower T. D. Mann R. V. (1978). The Jones fracture revisited. jbjs 60 (6), 776–782. 10.2106/00004623-197860060-00008 701310

[B15] Kibar B. Cavit A. Örs A. (2022). A comparison of intramedullary cannulated screws versus miniplates for fixation of unstable metacarpal diaphyseal fractures. J. Hand Surg.-Eur. 47 (2), 179–185. 10.1177/17531934211021521 34107786

[B16] Lam K. Bui R. Morris R. Panchbhavi V. (2021). Biomechanical analysis of conventional partially threaded screws versus headless compression screws in proximal fifth metatarsal (Jones) fracture fixation. Foot Ankle Spec. 14 (6), 509–514. 10.1177/1938640020931668 32506962

[B17] Lee K. T. Park Y. U. Young K. W. Kim J. S. Kim J. B. (2011). Surgical results of 5th metatarsal stress fracture using modified tension band wiring. Knee Surg. Sports Traumatol. Arthrosc. 19, 853–857. 10.1007/s00167-011-1406-3 21290105

[B18] Massada M. M. T. d.O. Pereira M. A. N. P. G. Sousa R. J. G. d. Costa P. G. Massada J. L. d.R. (2012). Osteossíntese com parafuso intramedular nas fraturas proximais do quinto metatarsiano do atleta. Acta Ortop. Bras. 20, 262–265. 10.1590/s1413-78522012000500003 24453614 PMC3718439

[B19] McKeon K. E. Johnson J. E. McCormick J. J. Klein S. E. (2013). The intraosseous and extraosseous vascular supply of the fifth metatarsal: implications for fifth metatarsal osteotomy. Foot Ankle Int. 34 (1), 117–123. 10.1177/1071100712460227 23386771

[B20] Mologne T. S. Lundeen J. M. Clapper M. F. O’Brien T. J. (2005). Early screw fixation versus casting in the treatment of acute Jones fractures. Am. J. Sports Med. 33 (7), 970–975. 10.1177/0363546504272262 15888715

[B21] Ochenjele G. Ho B. Switaj P. J. Fuchs D. Goyal N. Kadakia A. R. (2015). Radiographic study of the fifth metatarsal for optimal intramedullary screw fixation of Jones fracture. Foot Ankle Int. 36 (3), 293–301. 10.1177/1071100714553467 25253577

[B22] Orr J. D. Glisson R. R. Nunley J. A. (2012). Jones fracture fixation: a biomechanical comparison of partially threaded screws versus tapered variable pitch screws. Am. J. Sports Med. 40 (3), 691–698. 10.1177/0363546511428870 22227846

[B23] Porter D. A. (2018). Fifth metatarsal jones fractures in the athlete. Foot Ankle Int. 39 (2), 250–258. 10.1177/1071100717741856 29228800

[B24] Roche A. J. Calder J. D. (2013). Treatment and return to sport following a Jones fracture of the fifth metatarsal: a systematic review. Knee Surg. Sports Traumatol. Arthrosc. 21, 1307–1315. 10.1007/s00167-012-2138-8 22956165

[B25] Seidenstricker C. L. Blahous E. G. Bouché R. T. Saxena A. (2017). Plate fixation with autogenous calcaneal dowel grafting proximal fourth and fifth metatarsal fractures: technique and case series. J. Foot Ankle Surg. 56 (5), 975–981. 10.1053/j.jfas.2017.04.035 28606789

[B26] Sides S. D. Fetter N. L. Glisson R. Nunley J. A. (2006). Bending stiffness and pull-out strength of tapered, variable pitch screws, and 6.5-mm cancellous screws in acute Jones fractures. Foot Ankle Int. 27 (10), 821–825. 10.1177/107110070602701012 17054885

[B27] Singh S. K. Larkin K. E. Kadakia A. R. Hsu W. K. (2018). Risk factors for reoperation and performance-based outcomes after operative fixation of foot fractures in the professional athlete: a cross-sport analysis. Sports Health 10 (1), 70–74. 10.1177/1941738117729660 28915360 PMC5753966

[B28] Varner K. E. Harris J. D. (2017). The proximal fifth metatarsal metadiaphyseal jones fracture: intramedullary screw vs plantar plate. Oper. Tech. Sports Med. 25 (2), 59–66. 10.1053/j.otsm.2017.03.009

[B29] Wallace D. R. Shiver A. L. Pulliam S. K. Byrd B. M. McGee-Lawrence M. E. Snoddy M. C. (2023). Intramedullary threaded nail fixation versus plate and screw construct in metacarpal neck fractures: a biomechanical study. J. Am. Acad. Orthop. Surg. 31 (11), e516–e522. 10.5435/jaaos-d-22-00595 37071886 PMC10198952

[B30] Wheeler D. L. McLoughlin S. W. (1998). Biomechanical assessment of compression screws. Clin. Orthop. Rel. Res. 350, 237–245. 10.1097/00003086-199805000-00032 9602825

[B31] Willegger M. Benca E. Hirtler L. Kasparek M. F. Bauer G. Zandieh S. (2020a). Evaluation of two types of intramedullary Jones fracture fixation in a cyclic and ultimate load model. J. Orthop. Res. 38 (4), 911–917. 10.1002/jor.24530 31743452 PMC7155054

[B32] Willegger M. Benca E. Hirtler L. Moser L. Zandieh S. Windhager R. (2020b). Peroneus brevis as source of instability in Jones fracture fixation. Int. Orthop. 44, 1409–1416. 10.1007/s00264-020-04581-2 32372110 PMC7306048

[B33] Wright R. W. Fischer D. A. Shively R. A. Heidt R. S. Nuber G. W. (2000). Refracture of proximal fifth metatarsal (Jones) fracture after intramedullary screw fixation in athletes. Am. J. Sports Med. 28 (5), 732–736. 10.1177/03635465000280051901 11032233

[B34] Yamaguchi K. T. Telfer S. Iannuzzi N. Hoang D. Huang J. I. (2023). Ideal length and diameter for intramedullary screw fixation of metacarpal fractures: a biomechanical study. J. Hand Surg. Glob. Online 5 (2), 189–195. 10.1016/j.jhsg.2022.12.002 36974302 PMC10039306

[B35] Yates J. Feeley I. Sasikumar S. Rattan G. Hannigan A. Sheehan E. (2015). Jones fracture of the fifth metatarsal: is operative intervention justified? A systematic review of the literature and meta-analysis of results. Foot 25 (4), 251–257. 10.1016/j.foot.2015.08.001 26481787

